# Altered speech patterns in subjects with post-traumatic headache due to mild traumatic brain injury

**DOI:** 10.1186/s10194-021-01296-6

**Published:** 2021-07-23

**Authors:** Catherine D. Chong, Jianwei Zhang, Jing Li, Teresa Wu, Gina Dumkrieger, Simona Nikolova, Katherine Ross, Gabriela Stegmann, Julie Liss, Todd J. Schwedt, Suren Jayasuriya, Visar Berisha

**Affiliations:** 1 Department of Neurology, Mayo Clinic, Phoenix, AZ USA; 2 ASU-Mayo Center for Innovative Imaging, Phoenix, AZ USA; 3 School of Electrical, Computer and Energy Engineering and College of Health Solutions, Arizona State University, Tempe, AZ USA; 4 School of Industrial and Systems Engineering, Georgia Tech, Atlanta, GA USA; 5 School of Computing, Informatics, Decision Systems Engineering, Arizona State University, Tempe, AZ USA; 6 Phoenix VA Health Care System, Phoenix, AZ USA; 7 College of Health Solutions, Arizona State University, Tempe, AZ USA; 8 School of Arts, Media and Engineering, Arizona State University, Tempe, AZ USA

**Keywords:** Post-traumatic headache, Speech, mTBI, Language, Migraine

## Abstract

**Background/objective:**

Changes in speech can be detected objectively before and during migraine attacks. The goal of this study was to interrogate whether speech changes can be detected in subjects with post-traumatic headache (PTH) attributed to mild traumatic brain injury (mTBI) and whether there are within-subject changes in speech during headaches compared to the headache-free state.

**Methods:**

Using a series of speech elicitation tasks uploaded via a mobile application, PTH subjects and healthy controls (HC) provided speech samples once every 3 days, over a period of 12 weeks. The following speech parameters were assessed: vowel space area, vowel articulation precision, consonant articulation precision, average pitch, pitch variance, speaking rate and pause rate. Speech samples of subjects with PTH were compared to HC. To assess speech changes associated with PTH, speech samples of subjects during headache were compared to speech samples when subjects were headache-free. All analyses were conducted using a mixed-effect model design.

**Results:**

Longitudinal speech samples were collected from nineteen subjects with PTH (mean age = 42.5, SD = 13.7) who were an average of 14 days (SD = 32.2) from their mTBI at the time of enrollment and thirty-one HC (mean age = 38.7, SD = 12.5). Regardless of headache presence or absence, PTH subjects had longer pause rates and reductions in vowel and consonant articulation precision relative to HC. On days when speech was collected during a headache, there were longer pause rates, slower sentence speaking rates and less precise consonant articulation compared to the speech production of HC. During headache, PTH subjects had slower speaking rates yet more precise vowel articulation compared to when they were headache-free.

**Conclusions:**

Compared to HC, subjects with acute PTH demonstrate altered speech as measured by objective features of speech production. For individuals with PTH, speech production may have been more effortful resulting in slower speaking rates and more precise vowel articulation during headache vs. when they were headache-free, suggesting that speech alterations were related to PTH and not solely due to the underlying mTBI.

## Introduction

Individuals with migraine report changes in speech during migraine attacks and several studies have documented speech difficulty during the aura phase of the attack as well as prior to and during the attack [1–3]. Although severe alterations in speech are easily observed, objective methods for assessing subtle changes in speech production in subjects with headache remain sparse. A recent study that used a standardized speech production task demonstrated objective changes in speech patterns in people with migraine during the attack relative to healthy controls, as well as within-subject changes during the attack compared to between-attacks [4].

Post-traumatic headache (PTH) due to mild traumatic brain injury (mTBI) commonly has symptoms that are similar to those of migraine [5–7]. In fact, most subjects with PTH have migraine-like headache features [8]. However, there is a lack of investigations into whether speech changes are present in those with PTH. We hypothesized that individuals with PTH would have alterations in speech compared to healthy controls without history of mTBI. In order to determine speech changes in individuals with PTH, we used a speech elicitation task embedded within a mobile app to assess objective measures of speech that relate to a combination of motor and cognitive-linguistic components of speech. These include sentence speaking rate, pause rate during spontaneous speech production, pitch (average pitch and pitch variance), vowel and consonant articulation precision, and vowel space area. These measures were collected to: 1) investigate speech differences between individuals with PTH and healthy controls, and 2) assess whether individuals with PTH have speech changes during headaches compared to when they are headache-free.

The overarching goal of this study was to determine whether objective features measured from speech samples obtained from individuals with acute PTH could provide a surrogate measure of headache burden, which could have utility in the future for tracking headache persistence and recovery.

## Methods

This study received approval from the Mayo Clinic IRB in 2019. All subjects completed written informed consent. Subjects had to be native English-speakers aged 18–65 years. All subjects were required to have a mobile device with capability for downloading an application used to collect speech and had to be willing and able to provide a speech sample once every 3 days over a period of 3 months. Subjects with PTH were eligible for enrollment starting on the day of mTBI and until 59 days post-mTBI. Subjects had to meet criteria for acute PTH attributable to mTBI in accordance with the ICHD-3 criteria [9]. For individuals with PTH, history of headache or migraine was allowed. Healthy controls had to have no history of TBI and no history of three or more headaches per month. Exclusion criteria for subjects with PTH and healthy controls included the following: history of severe psychiatric disorder or neurological disorder (other than mTBI and headaches in the PTH group) and no history of a speech or language disorder. Study questionnaires: Subjects with PTH completed a detailed headache symptom questionnaire. All subjects completed the Ohio State University TBI Identification Method, a standardized questionnaire assessing the lifetime history of TBI for an individual (available at www.brainline.org) [10, 11], the Symptom Evaluation (step 2) of the Sports Concussion Assessment Tool (SCAT-5) questionnaire [12], the Beck Depression Inventory (BDI) [13] for assessing levels of depression, and the Delayed Auditory Verbal Learning Test (RAVLT, delayed recall, z-scored) for assessing verbal learning and memory [14]. The Symptom Evaluation of the SCAT-5 is a 22 item self-report of TBI symptoms. Participants rate each item based on how they feel on a 7-point Likert scale (0 (none) to 6 (severe)). Two totals are counted: total number of symptoms (0–22) and symptom severity (0–132). The BDI is a 21-item self-report to assess symptoms of depression. Each item is scored on a value of 0 to 3 and scores are combined for a total score (0–63). Scores between 0 and 13 identify no depression, 14–19 mild depression, 20–28 moderate depression and 29–63 severe depression.

For the RAVLT, a list of 15 words is read out loud by the examiner and the examinee is asked immediately afterward to recall as many words as they can. The list is read five times and each time, the examinee is asked to recall as many words from the list as they can, in any order. Next, a distractor list is read out loud, and the participant is asked to recall only the words from the distractor list. Afterward, the participant is then asked to recall only those words from the first list, which was read 5 times. After a delay of about 20 min (delayed recall), the examinee is asked again to recall as many words as possible from the first list. Only the delayed recall z-scores, which are a measure of episodic memory performance were included in this study.

At the first study visit, subjects were taught to download the speech application to their mobile devices. The study coordinator modeled the completion of speech elicitation tasks and the correct procedure for using the speech app. This included, selecting a time and place that is comfortable, without distractions and with minimal background noise. All subjects were asked to submit a speech sample every 3 days, beginning on the day of the first study visit and continuing over a period of the subsequent 12 weeks. As it was assumed that subjects with PTH would show the most significant speech changes during the acute phase of mTBI, therefore only the speech samples submitted during the first 30 days were used for comparison between subjects with PTH and healthy controls. When comparing subjects with PTH during headache to the headache-free phase, speech samples submitted over the first 90 days were used to increase the number of available samples.

### Speech assessment

The speech application was specifically developed for the objective evaluation of the following measures: sentence speaking rate, average pitch, pitch variance, vowel space area vowel and consonant articulation precision, and the spontaneous pause rate. As part of the speech app, subjects were asked to read out loud five sentences (sentence reading task) and to use spontaneous speech to describe activities of the previous day (spontaneous speaking task). The entire speech elicitation task took approximately 3 min to complete. Prior to starting the speech task, all subjects indicated whether they currently had a headache or whether they were headache free. If a current headache was reported then individuals were prompted to rate their headache intensity on a scale ranging from 1 (mild headache) to 10 (most severe headache imaginable). Table 1. shows a description of the speech measures that were extracted from the speech application.

**Table 1:**
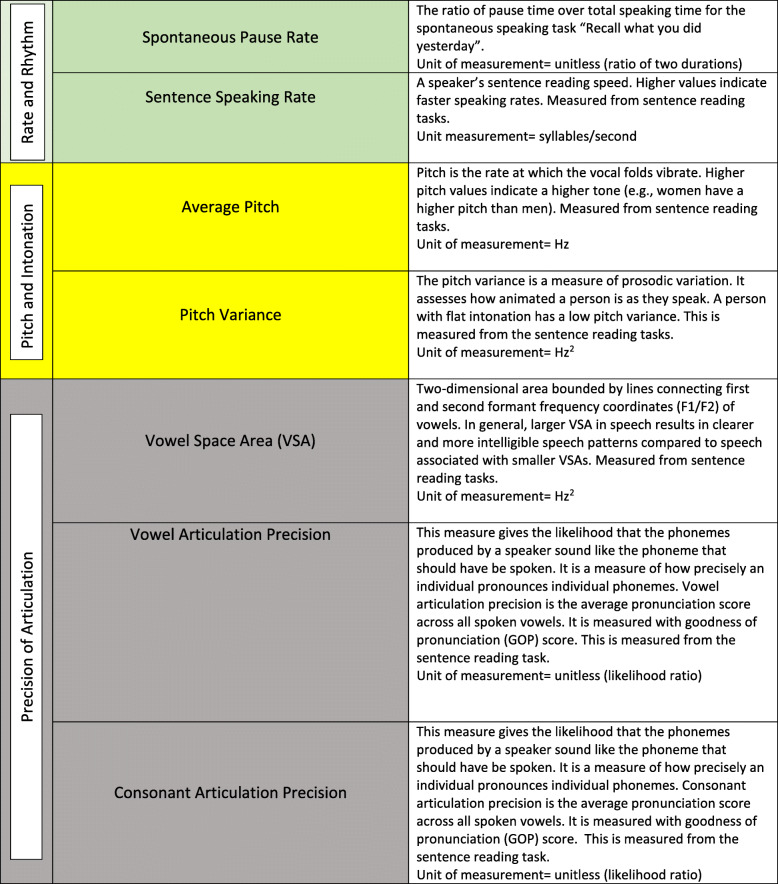
Description of speech features that were extracted from the speech application. The calculations for the sentence speaking rate, average pitch, pitch variance, vowel space area, vowel and consonant articulation precision were extracted from reading the following five sentences. 1. The supermarket chain shut down because of poor management. 2. Much more money must be donated to make this department succeed. 3. In this famous coffee shop, they serve the best donuts in town.4. The chairman decided to pave over the shopping center garden. 5. The standards committee met this afternoon in an open meeting.

### Speech features extraction and normalization

**Fig. 1:**
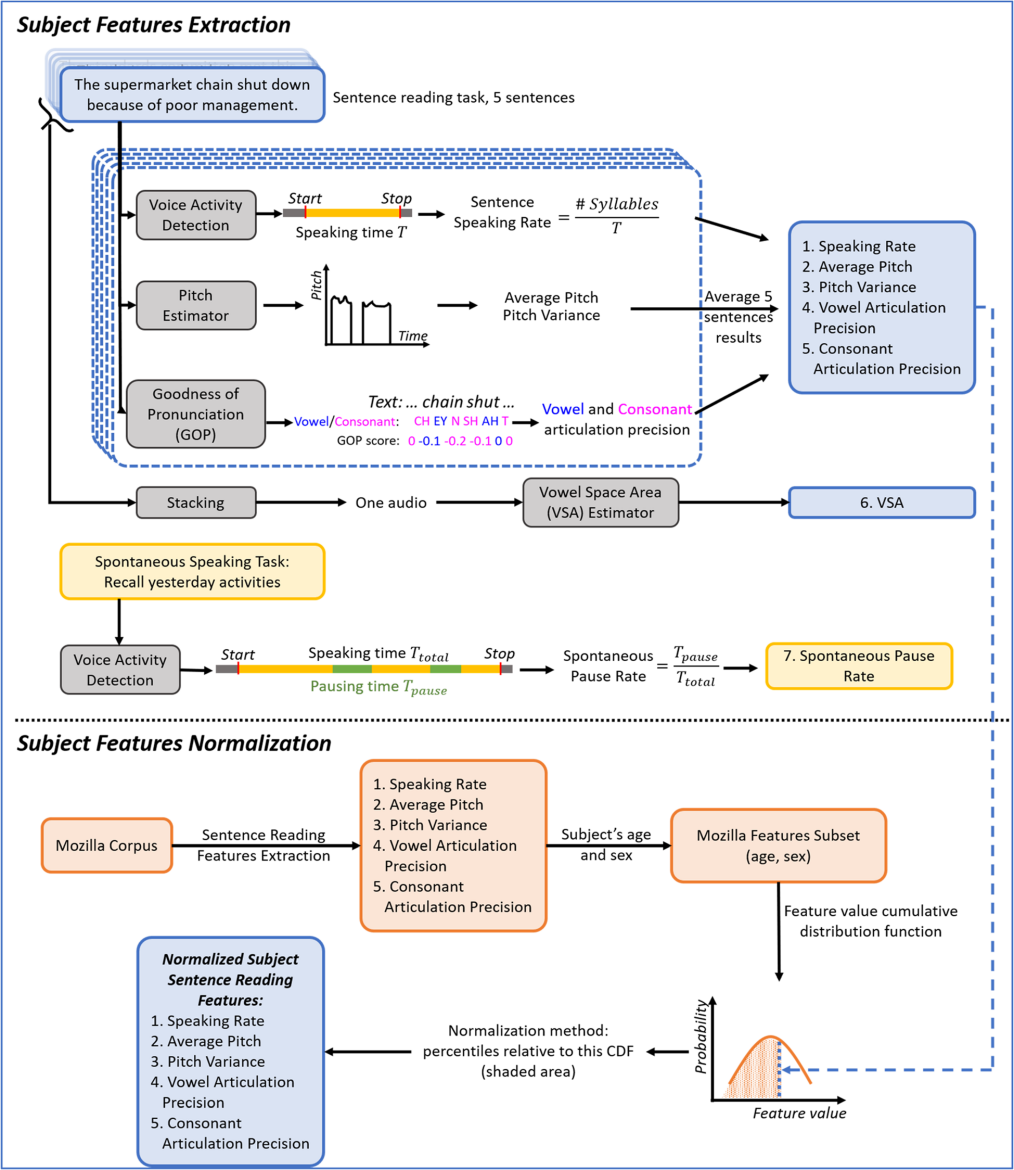
Speech feature extraction and normalization procedure for the sentence reading tasks and the spontaneous speaking task.

The methodology for extracting and normalizing speech features is shown in Fig. 1. After downloading the data from the server, the sentence reading audio samples (5 files) and spontaneous speaking audio sample (1 file) were used for speech feature extraction. The sentence speaking rate, average pitch, pitch variance, vowel and consonant articulation precision (measured with goodness of pronunciation scores) and vowel space area were extracted from the sentence reading tasks. The spontaneous pause rate was extracted from the spontaneous speaking task. The implementation details are described in the next section.

#### Sentence speaking rate

First, the total speaking time in a sentence audio sample was detected by using a Voice Activity Detection (VAD) algorithm [15] that identified the start and stop points of speech. The speaking rate was then calculated as the number of syllables (determined from the sentence reading prompt) divided by the speaking time. Consider the example of an individual reading the sentence “*the supermarket chain shut down because of poor management*” for 4.01 s. As there are a total of 15 syllables in the sentence: “*the su-per-mar-ket chain shut down be-cause of poor man-age-ment*”, the speaking rate was calculated as 15/4.01 = 3.74 syllables/second.

#### Pitch

The REAPER (https://github.com/google/REAPER) pitch estimator, was used to extract the pitch contour from the raw audio waveform for calculating the average pitch and pitch variance. The average pitch was estimated by calculating the sample mean from the pitch contour; similarly, the pitch variance was estimated by calculating the sample variance from the pitch contour.

#### Vowel and consonant articulation precision

The sentence reading audio files and corresponding sentence texts were estimated using the goodness of pronunciation (GOP) score evaluation algorithm [16] to generate the vowel and consonant articulation precision scores.

#### Vowel space area

All five sentence reading samples were concatenated into a continuous audio stream. The vowel space area was estimated using a extraction algorithm [17].

#### Spontaneous pause rate

**Fig. 2:**
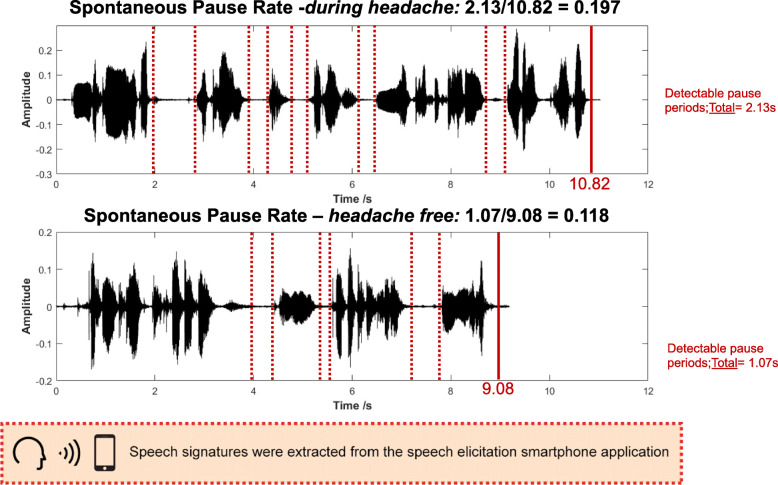
Example of the spontaneous pause rate differences when a person has a headache (top speech signature) as compared to when a person does not have a headache.

**Fig. 3:**
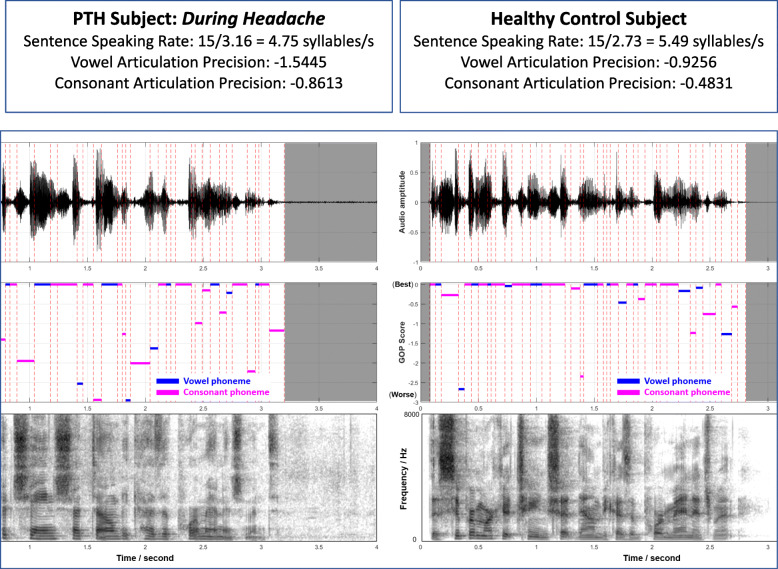
Example of sentence speaking rate differences, and vowel and consonant articulation precision differences of an individual with PTH *during headache* (left) as compared to Healthy Control (right) for the sentence reading task “The supermarket shut down because of poor management”, which contains 15 syllables and 40 phonemes.

The VAD algorithm was used to detect the timepoints during which the participant was speaking. The total speaking time was measured as the period from the speech start point to the speech stop point; the pause time was measured as the non-speech periods during spontaneous speech. The spontaneous pause rate was then calculated as the ratio of pause time over speaking time. For example, if a subject provided a spontaneous speech sample lasting 10.82 s seconds, and paused for 2.13 s during the task, then the spontaneous pause rate was calculated as 2.13/10.82 = 0.197. The pause rate was measured from the spontaneous speaking task. (See Figs. 2 and 3)

Since some speech features may depend on the age and sex of the speaker (e.g., older people typically speak more slowly and in lower pitch; female speakers generally have higher average pitch compared to male speakers), feature normalization was used to control for these potential confounding variables. Speech features were normalized by subject age and sex using the Mozilla common voice English database, a large open-source corpus for speech data [18]. This database contains sentence reading audio samples with corresponding texts for more than 11,000 individuals with age and sex demographics provided. Because it is a sentence reading database, only the features extracted from our sentence reading task are normalized, including sentence reading speaking rate, average pitch, pitch variance, and vowel and consonant articulation precision. Vowel space area is excluded from normalization because most individuals in the Mozilla database do not provide sufficient speech for computing this measure reliably. In addition, spontaneous pause rate is not normalized since there is no normative data for this task.

To normalize the features of a study participant, a nonparametric estimate of the cumulative distribution function (CDF) was computed from a subset of age/sex matched individuals in Mozilla. The features were converted to percentiles relative to this CDF and the normalized percentiles were then used as features.

### Statistical approach

Speech patterns of subjects with PTH and healthy controls were compared using a mixed-effects model with random (unique) intercepts for each participant and controlled for age and sex. The effect of healthy controls/PTH was tested on each speech measure. Age, sex, and group differences were treated as fixed effects. Differences on cohort demographics were assessed via two-sided *t*-tests or Fisher-exact tests, as appropriate.

Speech patterns between subjects with PTH during headache were compared to speech patterns of subjects with PTH when they were headache-free using a mixed-effects model with random (unique) intercepts for each participant and random (unique) slope for headache status. Age, sex, and headache status were modeled using fixed effects. The use of random slopes tests not only whether there was a *mean* difference in the metrics when subjects had a headache compared to when they were headache-free but it also tests the extent to which participants *differed* on their changes on speech measures (e.g., some participants might have very different speaking rates when they have a headache as compared to when they are headache-free, while others may not change much).

Therefore, a significant *p*-value may indicate mean differences in a measure when headache.

is present versus absent or indicate that participants vary in terms of how their scores differ when they have a headache or are headache-free. Given the limited sample size, the models for some speech metrics did not converge. Non-convergence occurs when the model is too complex, the sample size is too small, or the model is not supported by the data, which results in the model being unable to reach a stable solution. Therefore, only the models that converged are reported.

## Results

Nineteen subjects with PTH (mean age = 42.5, SD = 13.7; 13 females, 6 males) and 31 healthy controls (mean age = 38.7 SD = 12.5, 18 females, 13 males) participated. There were no significant differences between groups for age (*p* = 0.32) or sex (*p* = 0.55); see Table 2, *Subject Demographics*. Among those with PTH, 9 had mTBI that were due to motor vehicle accidents, 3 that were due to falls, 5 that were due to sports-related injuries, and 2 that were due to hitting their head in home-related accidents. Fifteen subjects with PTH had no prior mTBI, 1 subject had one prior mTBI, and 3 subjects had two prior mTBIs. Twelve subjects reported no loss of consciousness, and 7 had loss of consciousness. As part of completing the headache questionnaire, individuals reported medication use for treating headache. Nine individuals reported treating headache with NSAIDs, and ten patients did not treat headache with medication. There were significant differences between groups on the symptom assessment of the SCAT-5, with individuals with PTH reporting more severe symptoms (symptom assessment total score: PTH = 28.47; SD = 26.7; healthy controls = 1.97, SD = 4.0; *p* < 0.001) and they had significantly lower delayed recall z-scores compared to healthy controls (delayed recall: PTH = − 0.9, SD = 1.1; healthy controls = .15, SD = 1.2; *p* = 0.004). Symptoms of aura including difficulty with speech was only reported by one individual with PTH. Although there were significant group differences on raw scores of the BDI, the mean raw scores of both groups were in the ‘normal, non-depressed’ range. On average, subjects with PTH were seen two-weeks post-mTBI (14.8 days, range 4–42 days).

**Table 2:** Subject Demographics. Average headache pain = ranges from 1 to 10 (1 = mild headache to 10 = worst headache imaginable) BDI=Beck Depression Inventory, mean raw score; SCAT-3 = Sport concussion assessment tool (SCAT-5), Symptom Evaluation, total score; RAVLT delayed recall = Ray Auditory Verbal learning test, delayed recall, z-score; Days post-mTBI = number of days between the date of mild traumatic brain injury and the date of the baseline visit, on average, patients with PTH were seen two-weeks post-concussion (14.8 days, range 4–42 days); HC = healthy controls; n/a = not applicable; PTH=Post-traumatic Headache

	PTH (***n*** = 19)	HC (***n*** = 31)	PTH vs HC ***p***-value
Age, mean (SD)	42.5 (13.7)	38.7 (12.5)	0.32
Sex female/male	13/6	18/13	0.55
BDI mean (SD)	7.63 (5.6)	2.06 (3.5)	0.001*
Symptom Evaluation; *total*	28.47 (26.7)	1.97 (4.0)	< 0.001
RAVLT delayed recall	−.90 (1.1)	.15 (1.2)	0.004
Average headache pain (1–10)	4.53 (2.1)	n/a	n/a
Days post mTBI	14.8 (32.2)	n/a	n/a

*Although there were statistically significant group differences between the BDI raw scores, the average raw scores of each group was in the ‘normal’, i.e. non-depressed range, according to the BDI scoring criteria

A total of 1122 speech samples were collected (healthy controls = 622; subjects with PTH = 500; 180 samples of PTH during headache and 320 samples of PTH without headache).

### Speech differences between individuals with PTH and healthy controls

Regardless of headache presence or absence, individuals with PTH had significantly reduced consonant precision (not normalized: *p* = 0.008; normalized: *p* = 0.0015) and vowel precision (not normalized: *p* = 0.007; normalized: *p* = 0.0368) and longer pause rates (0.0098) relative to healthy controls. On days when PTH subjects had headache, subjects had significantly longer pause rates (*p* = 0.0043), slower sentence speaking rates (not normalized: *p* = 0.0369; normalized: *p* = 0.0137) and less precise vowel (not normalized: *p* = 0.049; normalized vowel articulation was not significant: *p* = 0.1948) and consonant articulation (not normalized: *p* = 0.0028; normalized: *p* = 0.0038) compared to healthy controls.

Table 3 and 4. show the speech measures, the *p*-values for the healthy controls/PTH differences (p-value computed based on a Chi Squared Likelihood Ratio Test), the mean values (e.g., mean pause rate) for the two groups, and the difference between the two groups. A significant *p*-value indicates that the mean speech measure differed significantly between the control and PTH groups.

**Table 3:**
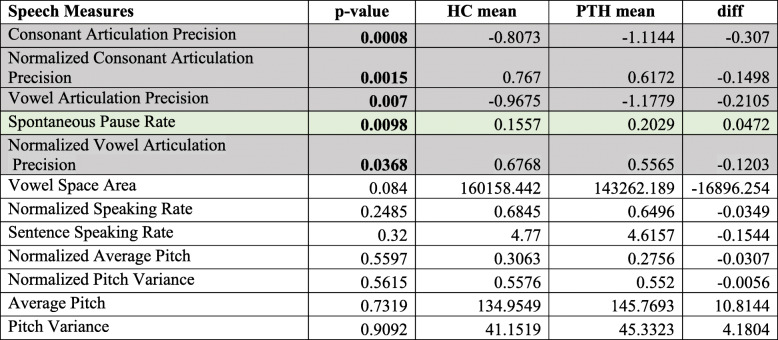
Differences in speech patterns between patients with PTH and Healthy Controls. diff = differences between the means of speech measures between PTH and HC, for assessing the direction of differences only. Negative numbers indicate lower values, and positive numbers indicate higher values for patients with PTH. Bolded *p*-values indicate significant differences between PTH and HC. Normalized = the value is normalized by a large corpus (for details refer to Table 1).

Speech features that are significantly different between groups are color-coded for easy cross-referencing with speech features shown in Table 1

**Table 4:**
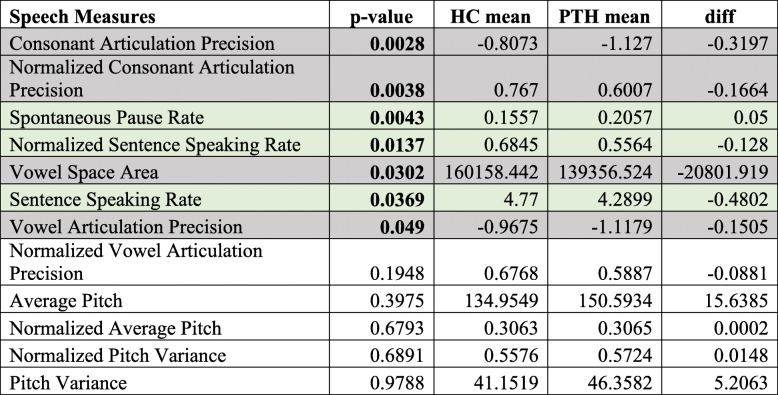
Differences in speech patterns between HC and PTH patients *during headache.* Results of linear mixed model with individuals as random effects, controlled for age and sex.

Bolded *p*-values indicate significant differences between PTH and HC; diff = differences between the means of speech measure between PTH patients *during headache* and HC, for assessing the direction of differences only. Negative numbers indicate lower values, and positive numbers indicate higher values for patients with PTH *during headache*

Normalized = the value is normalized by a large corpus (for details refer to Table 1)

Speech features that are significantly different between groups are color-coded for easy cross-referencing with Speech Features shown in Table 1

The means and differences are based on the sample cohorts and not based on the mixed-effects model and are provided in order to give context to the p-value and to evaluate the directionality of the effect.

### Speech differences in individuals with PTH during headache compared to the headache-free state

During headache, PTH subjects had significantly slower sentence speaking rates (not normalized: *p* = 0.002; normalized: *p* < 0.0001) but more precise vowel articulation (normalized: *p* = 0.0052) compared to when they were headache-free. Table 5. shows the speech measures, the *p*-values for the differences between the headache states, the mean values for the two headache states, and the mean differences between the two headache states. The means and differences are based on the raw scores of the sample and not based on the mixed-effects model and are provided in order to give context to the *p*-values and to evaluate the directionality of the effect. Two sets of p-values are provided: p-values for the random-intercepts models and p-values for random-intercepts-random-slopes models. As previously explained, significant p-value in the random-intercepts model indicates that the mean speech measure differed significantly between the headache states, while a significant p-value in the random-intercepts-random-slopes indicates significant mean differences and between-participant variability in the differences between the two states.

**Table 5:**
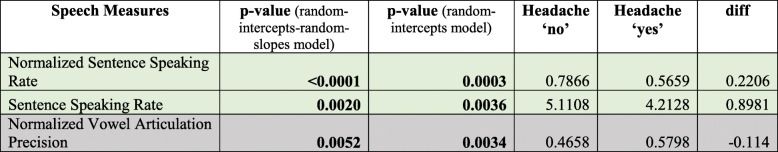
Speech pattern differences in patients with PTH during days of headache (headache ‘yes’) compared to days of headache freedom (headache ‘no’). Results of linear mixed model with individuals as random effects controlled for age and sex. Random-intercepts model = Bolded p-values indicate that the mean of the speech measure is different when the participant has a headache as compared when she/he is headache free (within-person effect). Random-intercepts-random-slopes model = Bolded p-values indicate that there were differences not only on the mean of the speech measures between the headache states but also on how participant differed on their changes on speech measures between the two headache states (within- and between- person effects).

Speech features that are significantly different between groups are color-coded for easy cross-referencing with Speech Features shown in Table 1

## Discussion

The results of this study demonstrate longer pause rates, slower sentence speaking rates and less precise vowel and consonant articulation in patients with PTH during headache compared to healthy controls as well as slower sentence speaking rates and altered vowel articulation in individuals with PTH *during headache* as compared to when PTH subjects were headache-free.

Our results are in agreement with previous migraine and chronic pain studies which identified slower motor speech production (speech alternating motion rates) in individuals with chronic back pain [19] and changes in speaking rate, articulation rate, articulatory precision, phonatory duration, and intonation shown between individuals with migraine relative to healthy controls as well as within the migraine group during the pre-attack vs. attack vs. interictal periods [4]. Although it was not the focus of the current study, it is important to note that psycholinguistic changes are also observed in patients suffering from psychological trauma, such as post-traumatic stress disorder [20] and childhood trauma [21, 22]. Although these disorders are difficult to disentangle (i.e., PTSD and PTH due to TBI often co-occur), future studies are needed to assess the similarities and unique differences of speech alterations in patients with PTH from speech alterations in individuals with PTSD from changes in speech in children suffering emotional distress due to traumatic life experiences.

### Speech rate and rhythm (spontaneous pause rate and sentence speaking rate)

Compared to healthy controls, individuals with acute PTH demonstrated alterations in speech rate and rhythm (i.e., longer pause rates and slower sentence speaking rates). There is emerging data that individuals with PTH have difficulty understanding and performing cognitive-linguistics tasks, have difficulty understanding and processing rapid speech [23] and show electrophysiological evidence of abnormal auditory processing [24]. Saunders et al., found that blast-exposed veterans with mTBI had auditory processing deficits despite having clinically normal hearing [25], and Strockbridge and colleagues found that concussed children had altered language profiles and difficulty with semantic and syntactic access relative to non-concussed healthy children [26]. Furthermore, a recent study by Talkar et al. showed that vocal acoustic features of articulation, phonation and respiration can distinguish individuals with subclinical mTBI from healthy controls [27].

Although not the focus of this study, participants with acute PTH did show significantly worse performance on a delayed word recall task (RAVLT, delayed recall) and more cognitive, behavioral and mood related symptoms (SCAT-5). Therefore, pause rates in individuals with PTH could be an indication of word-finding difficulties and may serve as a proxy for cognitive function in individuals with PTH. However, future studies are needed that specifically relate post-mTBI symptoms including cognitive function to changes in speech.

### Precision of articulation (vowel space area, vowel and consonant articulation precision)

In the current study, individuals with PTH during headache also showed alterations in the precision of articulation, specifically reduced vowel space area relative to healthy controls.

Vowel space area is an acoustic metric commonly used for measuring articulatory function. Previous data have shown reduced vowel space area in patients with motor speech disorders [28] including Parkinson’s disease and cerebral palsy as well as in patients with depression and those suffering from post-traumatic stress disorder and vowel space area has thus been suggested as a potential marker for psychological distress [29]. In the current study, subjects had depression symptoms within normal/healthy range, therefore we posit that reduced vowel space area may be a manifestation of either speech production under stress (i.e., headache pain intensity) or related to difficulties with speech-motor control due to the underlying mTBI. This hypothesis is further supported by the reductions in both vowel and consonant precision in patients with PTH relative to healthy controls and the reduction in speaking rate between the PTH group and the HC group and between PTH during headache vs when individuals with PTH were headache free.

The disruption in speech pattern in subjects with PTH might be a result of brain structural or functional changes in auditory and language pathways such as the posterior thalamic fasciculus and the superior and inferior longitudinal fasciculus. However, the neural underpinnings of speech changes will need to be further interrogated by associating brain structural and functional data with speech features in subjects with PTH.

Individuals with PTH had more precise vowel articulation during headache compared to when they were headache-free. It may be hypothesized that during headache, when speech production requires more effort (hence resulting in slower speaking rates), individuals need to pay more attention to the production of speech and thus paradoxically produce more precise vowel articulation.

### Limitations

It is possible that several factors may have influenced individuals’ speech patterns and introduced variance to our result including 1) mTBI mechanism (sports-related vs, motor vehicle accident vs fall), or 2) the number of previous mTBIs. Additionally, future studies are needed that assess speech features in subjects with mTBI without headache to subjects with PTH to specifically disentangle speech changes due to mTBI from speech changes due to headache. Additionally, future studies are needed to isolate speech alterations in individuals with PTH without history of PTSD from individuals who suffer from PTSD without history of mTBI. In the current study, the model for pause rate did not converge in the within-subject analysis which is likely due to the relatively small sample size in the study. We posit that a larger study, with more speech samples captured during periods of headache and no-headache per individual, would further show that reductions in pause rate are apparent in the within-subject analysis as well.

## Conclusion

Our results indicated changes in speech rate and rhythm and alterations in precision of articulation in individuals with PTH to due mTBI relative to healthy controls as well as a reduction in sentence speaking rate and alterations in vowel articulation precision when individuals with PTH had a headache compared to when they were headache-free -- potentially suggesting that PTH-related pain can modify healthy speech patterns. Currently, there is not a way to predict when and whether an individual with PTH will recover. The current results indicate that speech detection using a speech application downloaded on a mobile device might be a practical, objective, and early rapid screening tool for assessing headache-related burden and may have potential for predicting headache recovery in subjects with acute PTH. Additionally, the recognition of speech changes in individuals with acute PTH could be important for identifying those individuals at ‘high risk’ for developing persistent post-traumatic headache and may allow physicians to begin headache treatment early, when it might be most effective, in order to prevent headache chronification.

### Key findings

- Relative to healthy controls, individuals with acute PTH show aberrations in objective speech features.
- Speech changes are exacerbated in PTH subjects during headache.
- Speech pattern analysis might have utility for assessing headache burden and recovery.

## Data Availability

Researchers wishing to access our data should send their request via e-mail to the corresponding author (chong.catherine@mayo.edu) and the Mayo Clinic Institutional Review Board (IRBE@mayo.edu).

## Abbreviations

BDI: Beck depression inventory
CUD: Cumulative distribution function
GOD: Goodness of pronounciation
HC: Healthy controls
mTBI: Mild traumatic brain injury
PTH: Post-traumatic headache
SD: Standard deviation
TBI: Traumatic brain injury
VAD: Voice activity detection
